# People reporting experiences of mediumship have higher dissociation symptom scores than non-mediums, but below thresholds for pathological dissociation

**DOI:** 10.12688/f1000research.12019.3

**Published:** 2018-01-04

**Authors:** Helané Wahbeh, Dean Radin

**Affiliations:** 1 Institute of Noetic Sciences, Petaluma, CA, 94952, USA

**Keywords:** Dissociation, Anomalous information reception

## Abstract

**Background: **Dissociative states exist on a continuum from nonpathological forms, such as highway hypnosis and day-dreaming, to pathological states of derealization and depersonalization. Claims of communication with deceased individuals, known as mediumship, were once regarded as a pathological form of dissociation, but current definitions recognize the continuum and include distress and functional disability as symptoms of pathology. This study examined the relationship between dissociative symptoms and mediumship in a large convenience sample.

**Methods: **Secondary analyses of cross-sectional survey data were conducted. The survey included demographics, the Dissociation Experience Scale Taxon (DES-T, score range 0-100), as well as questions about instances of mediumship experiences. Summary statistics and linear and logistic regressions explored the relationship between dissociative symptoms and mediumship endorsement.

**Results: **3,023 participants were included and were mostly middle-aged (51 years ± 16; range 17-96), female (70%), Caucasian (85%), college educated (88%), had an annual income over $50,000 (55%), and were raised Christian (71%) but were presently described as Spiritual but not Religious (60%). Mediumship experiences were endorsed by 42% of participants, the experiences usually began in childhood (81%), and 53% had family members who reported similar experiences. The mean DES-T score across all participants was 14.4 ± 17.3, with a mean of 18.2 ± 19.3 for those claiming mediumship experiences and 11.8 ± 15.2 for those who did not (t = -10.3, p < 0.0005). The DES-T threshold score for pathological dissociation is 30.

**Conclusions: **On average, individuals claiming mediumship experiences had higher dissociation scores than non-claimants, but neither group exceeded the DES-T threshold for pathology. Future studies exploring dissociative differences between these groups may benefit from using more comprehensive measures of dissociative symptoms as well as assessments of functional impairment, which would help in discerning between pathological and non-pathological aspects of these experiences.

## Introduction

Dissociation is conceptualized as the disruption of usually integrated functions of consciousness, memory, identity or perception of the environment ^1^. Dissociative Identity Disorder (DID) is defined as a personality disorder, when two or more distinct identities or personalities are present, each with its own pattern of perceiving, relating to or thinking about the environment and self. The core clinical symptoms of DID include amnesia, depersonalization, derealization, identity confusion and identity alteration. Dissociative states are prevalent in other psychiatric disorders, such as PTSD ^2^, and are more prevalent in younger nonclinical populations ^3^. Dissociative states exist on a continuum ^4– 6^, from nonpathological expressions, such as highway hypnosis and day-dreaming, to pathological states of derealization (surrealness), and depersonalization (absence of identity) ^7^. Almost half of United States adults have experienced a dissociative episode at some time in their lives ^8^.

A widespread belief possibly related to dissociative symptoms is the idea that it is possible to communicate with deceased individuals; people who report such experiences are called “mediums” ^9^. A survey of 18,607 people in the United States and thirteen European countries found that some 25% reported contact with the dead ^10^. Double-blind experiments indicate that in some cases the information obtained by mediums can be verified as accurate ^11– 14^. This suggests that claims of mediumship experiences should not be uniformly dismissed as pathological, however, such claims are commonly regarded as symptoms of DID ^5, 15, 16^. This is despite a lack of clear evidence that mediums exhibit greater pathological symptoms than the general population ^17, 18^. Perhaps this is because on average, mediums do not regard dissociative symptoms as distressful. Indeed, the most recent Diagnostic and Statistical Manual of Mental Disorders (5 ^th^ edition) clarifies that pathological DID is defined when “the person must be distressed by the disorder or have trouble functioning in one or more major life areas because of the disorder,” and that “the disturbance is not part of normal cultural or religious practices” ^1^.

In an effort to further our understanding of the relationship between self-report dissociative symptoms and claims of mediumship, we analyzed data from a large convenience sample. We hypothesized that the prevalence of dissociative symptoms in people who claim mediumship abilities would be the same as those who do not maintain such claims.

## Methods

This study includes secondary analyses of a specific subset of data on mediumship experiences and dissociative symptoms collected for a different research study approved by the Institute of Noetic Sciences (IONS) Institutional Review Board (approval number, wahh_2016_01). A survey was administered through SurveyMonkey.com with HIPAA compliant methods. Participants were recruited through the IONS Facebook page, IONS mailing lists, and the IONS community networks.

The survey for the parent study from which the data for this study was extracted ( Supplementary File 1) began with the study’s purpose and informed consent details. Date and country of birth, race, education, and childhood and current spiritual/religious affiliation were collected. Gender was collected on a subsample of participants. Participants indicated if they had mediumship experiences, defined as the “ability to mediate communication between spirits of the dead and the living or the empathic ability to feel the presence and energies of spirits.” They also indicated age of onset (if applicable), and family history of similar experiences.

### Dissociation Measure

Participants then completed the Dissociation Experiences Scale Taxon (DES-T) ^3^ that can be used to distinguish pathological from non-pathological dissociation with a threshold score of 30 with an 87% positive predictive value (Cronbach Alpha of 0.75, on a scale of 0–100) ^19, 20^. The DES-T is just one of many dissociative symptom instruments and was chosen for this study because of its brevity and common use. Respondents selected a percent frequency for eight dissociative symptoms. The DES-T results in two variables: the mean of the eight items; and a binary variable based on the pathology threshold ^3^.

### Statistical analysis

Categorical variable percentages were calculated and examined qualitatively. Means, standard deviations and ranges of continuous variables were calculated. Covariates included gender, age, race, education, income, childhood spirituality and current spirituality, family history, and age of the claimed ability onset. Missing values were randomly distributed except for gender. T-test and chi-square tests evaluated differences between variables. Linear and logistic regressions examined the relationship between dissociative symptoms scores and mediumship experience status. A Bonferroni multiple comparison correction was applied to the α significance value, designating 0.003 as the cutoff for a significant result (α = 0.05 divided by 19 items, including seven demographic items, eight DES-T items, DES-T total, DES-T cut-off, linear and logistic regression. Statistics were performed using Stata 12.0 (StataCorp LLC, College Station, Texas).

## Results

In total, 3984 participants took the survey from May 4, 2016 to June 7, 2017. Participants were not required to complete all fields and thus only data from 3023 participants who answered the mediumship question (question 49 of the survey) and completed the DES-T (question 75) were included. Most participants were from the United States (62.6%), followed by the United Kingdom (7.7%) and Canada (6.3%); the remaining participants represented thirteen other countries. Participants were mostly middle aged (51 years ± 16; range 17-96), female (70%), Caucasian (85%), college educated (88%), had an annual income over $50,000 (55%), were raised Christian (71%), and now affiliated as Spiritual but not Religious (60%; Table 1). Current spiritual/religious affiliation was different by mediumship status.

**Table 1.** Demographic variables for participants by purported ability for anomalous information reception about deceased humans. Mean ± standard deviation; t, Student’s two-sample t-test statistic; X ^2^, chi-square statistic; p, probability.

	Mediumship				
	Yes N - 1,257	No N - 1,766	N	*t* or *X ^2^*	*p*
**Age**	51.7 ± 14.3	51.4 ± 16.4	2751	−0.4	0.68
	Range (17–96)	Range (17–89)			
**Gender (% Female)**	80.0%	67.2%	519	5.99	0.01
**Race (% Caucasian)**	86.5%	83.6%	2970	4.76	0.03
**Education (% ≥ some college)**	87.3%	88.9%	2977	1.66	0.20
**Income (% ≥ $50,000 annual income)**	38.6%	35.7%	2768	2.32	0.13
**Childhood Spiritual/Religious** **Affiliation (% Christian)**	71.7%	70.6%	2986	0.44	0.51
**Current Spiritual/Religious Affiliation** **(% Spiritual but not religious)**	65.9%	56.1%	2991	29.6	<0.0005*

Mediumship experiences were endorsed by 42% of participants, with their first experience starting in childhood (81%), and 53% having family members with similar experiences. The grand mean DES-T score was 14.4 ± 17.3 (range 0–100) across all participants and was significantly higher for mediumship claimants (18.2 ± 19.3) than for non-claimants (11.8 ± 15.2; t = -10.3, *p*<0.0005; Table 2). A linear regression model for the DES-T total score and mediumship responses, including all covariates, found race and education to be significant predictors (F (3, 2947) = 73.2, p<0.0005). Some 11% of mediumship non-claimants and 22% of mediumship claimants had scores greater than 30 (X ^2^ = 63.0, p<0.0005). A logistic regression based on this threshold showed a significant difference in mediumship responses with education (> college) and income (>$50,000) to be significant covariates (LR X ^2^ = 99.12, p< 0.0005).

**Table 2.** The eight item and total means, standard deviations, and mean difference sorted by highest mean percentage by mediumship endorsement. Data are presented as the mean ± standard deviation. DES-T, Dissociation Experiences Scale Taxon; t - Student’s two-sample t-test statistic; p, probability.

	Mediumship				
DES-T Item	Yes (n=1257)	No (n=1766)	Mean Difference	t	p
5. Some people sometimes have the experience of feeling that other people, objects, and the world around them are not real.	25.8 ± 32.0	17.5 ± 26.7	8.3	-7.7	<0.0005*
8. Some people sometimes find that they hear voices inside their head which tell them to do things or comment on things that they are doing.	25.5 ± 33.5	13.7 ± 25.7	11.8	-11.0	<0.0005*
3. Some people sometimes have the experience of feeling as though they are standing next to themselves or watching themselves do something and they actually see themselves as though they were looking at another person.	22.2 ± 29.8	12.9 ± 22.9	9.3	-9.7	<0.0005*
7. Some people find that in one situation they may act so differently compared to another situation that they feel almost as if they were two different people.	21.5 ± 30.6	17.6 ± 28.2	3.9	-3.6	<0.0005*
6. Some people sometimes have the experience of feeling that their body does not seem to belong to them.	20.6 ± 30.0	13.4 ± 24.5	7.2	-7.2	<0.0005*
1. Some people have the experience of finding themselves in a place and having no idea how they got there.	13 ± 23.3	7.8 ± 17.2	5.2	-7.06	<0.0005**
2. Some people have the experience of finding new things among their belongings that they do not remember buying.	10.4 ± 21.5	6.9 ± 17.4	3.5	4.95	<0.0005
4. Some people are told that they sometimes do not recognize friends or family members.	6.6 ± 17.7	4.3 ± 14.3	2.3	-4.0	.0001*
**Total**	18.2 ± 19.3	11.8 ± 15.2	6.4	10.3	<0.0005*

Dissociation symptoms for those with and without self-report anomalous information reception DT# are the Dissociation Experience Scale Taxon items. Click here for additional data file. Copyright: © 2018 Wahbeh H and Radin D 2018 Data associated with the article are available under the terms of the Creative Commons Zero "No rights reserved" data waiver (CC0 1.0 Public domain dedication).

## Discussion

In total, 42% of participants endorsed mediumship experiences. Given that the population surveyed was comprised mostly of individuals interested in mediumship-type experiences, this high percentage is not surprising. The prevalence of similar “contact with the dead” reports in other surveys ranges from 10% ^21^, 25–30% ^10^, 29% ^22^, and up to the same figure found in the present survey, 42% ^23^. The overall mean dissociation experience score for mediumship claimants was substantially below the DES-T clinical cutoff for pathological dissociation, but it was significantly higher than for non-claimants ^3, 24^.

The threshold for determination of pathological dissociation continues to be debated, and the present findings may be idiosyncratic with respect to use of the DES-T scale ^3, 24, 25^. In addition, the experience of communicating with the dead may also be considered a symptom of a high degree of schizotypy, not just dissociation ^26^. We also note that the grand mean DES-T score in our sample was higher than that observed in the general population ^19^. This again likely reflects the convenience sampling of IONS members, which reduces the generalizability of our findings. Future studies comparing those who claim versus do not claim mediumship experiences may benefit from use of more comprehensive measures of dissociative symptoms. In addition, specifically asking questions about functional impairment would help discern between pathological and nonpathological aspects of purported mediumship experiences.

## Data availability

The data referenced by this article are under copyright with the following copyright statement: Copyright: © 2018 Wahbeh H and Radin D

Data associated with the article are available under the terms of the Creative Commons Zero "No rights reserved" data waiver (CC0 1.0 Public domain dedication). 

Dataset 1: Dissociation symptoms for those with and without self-report anomalous information reception. DT# are the Dissociation Experience Scale Taxon items. doi, 10.5256/f1000research.12019.d171352 ^26^

## Supplementary material

Supplementary File 1: Survey on genetics of psychic ability.
