# Supplementary material for: People reporting experiences of mediumship have higher dissociation symptom scores than non-mediums, but below thresholds for pathological dissociation
Source: F1000Res. 2018 Jan 4;6:1416. Originally published 2017 Aug 10. [Version 3] doi: 10.12688/f1000research.12019.3 (PMC5782403; doi:10.12688/f1000research.12019.3)
Supplement: Supplementary file 2 [file f1000research-6-14736-s0001.tgz › aa70b2f8-6e41-4939-a370-9da9ea3a5276.pdf]

<u>TITLE:</u> Genetics of Psychic Ability

<u>PRINCIPAL INVESTIGATOR:</u> Helané Wahbeh, ND, MCR 707-779-8230

<u>FUNDED BY:</u> BIAL and Institute of Noetic Sciences. For more information about the Institute of Noetic Sciences, please go to www.noetic.org

<u>PURPOSE:</u> You have been invited to be in this research study because you have expressed interest in contributing information about the genetics of extended human capacities. The purpose of this study is to discover if extended human capacities run in families, suggesting a genetic component.

<u>PROCEDURES:</u> This online survey will take approximately 20-30 minutes to complete. In this survey, you will answer questions about yourself and your family. You will be asked about your gender, marital status; extended human capacities that you or your family may have; medications you may be taking; and personal experiences you may have had. At the end of the survey you will be asked if we have permission to contact you for the next phase of this research project.

If you have any questions, concerns, or complaints regarding this study now or in the future, please contact Dr. Helané Wahbeh, 707-779-8230.

<u>RISKS:</u> We make every effort to protect your identity, however there is a minimal risk of loss of confidentiality. If you find some of the questions too personal or or wish not to answer you may leave them blank.

<u>BENEFITS:</u> You may or may not find personal benefit from being in this study. However, by serving as a participant you will help us learn how to benefit others in the future.

<u>CONFIDENTIALITY:</u> In this study we are receiving minimal identifiable information about you so there is little chance of breach of confidentiality.

<u>COSTS:</u> There is no cost for participating in this study.

<u>PARTICIPATION:</u> This research is being overseen by the Institute of Noetic Science (IONS) Institutional Review Board (“IRB”). If any of the follow apply, please contact the IRB Chair, Dr. Dean Radin, at dean@noetic.org.

- **Your questions, concerns, or complaints are not being answered by the research team.**
- **You want to communicate with someone other than the research team.**
- **You have questions about your rights as a research subject.**
- **You want to get more information or provide input about this research.**

You are not required to participate in this or any research study. You are free to withdraw from the study at any time.

Thank you for your participation!

Demographics

1. What is your date of birth?

	month	day	year
month/day/year			

2. What time were you born (if known)?

3. In what city were you born?

4. If in US, what state or U.S. territory were you born?

5. What is your country of birth?

6. Which race/ethnicity best describes you?

7. What is the highest level of education you have completed?

8. Which of the following best describes your current occupation?

9. What is your approximate average household income?

10. What is your marital status?

11. What was your primary childhood spiritual/religious affiliation? (pick one)

12. On a scale of 0 (not at all) to 5 (deeply) how much did religion or spirituality influence your upbringing? In other words, how much was religion or spirituality a part of your family life while growing up?

13. What is your primary current spiritual/religious affiliation?

14. How important is your religious or spiritual practice to you now?

Animal Communication Ability

Directions: You will now be presented with unique abilities or experiences some people have had. Please mark YES or NO if you have the ability or not. If you mark YES, you will be asked to rate how strong and accurate the ability is and how often you experience it.

15. Animal Communication - The empathic ability (beyond the five physical senses) to hear, feel and communicate with animals.

- ○ Yes
- ○ No

Animal Communication Rating

16. Please rate your Animal Communication ability.

On a scale of low (left) to high (right)....
Strength -How intensely do you experience this ability?
Accuracy- How correct is the information?
Frequency- How often do you experience this ability?

	Weak/Inaccurate/Rarely				Strong/Accurate/Often
Strength	◯	◯	◯	◯	◯
Accuracy	◯	◯	◯	◯	◯
Frequency	◯	◯	◯	◯	◯

Aura Reading Ability

17. Aura Reading- Perception of energy fields surrounding people, places and things.

◯ Yes

◯ No

Aura Reading Rating

18. Please rate your Aura Reading ability.

On a scale of low (left) to high (right)....
Strength -How intensely do you experience this ability?
Accuracy- How correct is the information?
Frequency- How often do you experience this ability?

	Weak/Inaccurate/Rarely				Strong/Accurate/Often
Strength	○	○	○	○	○
Accuracy	○	○	○	○	○
Frequency	○	○	○	○	○

Automatic Writing Ability

19. Automatic Writing or Psychography- Writing produced without conscious thought, produced by or under the influence of a spirit.

- ◯ Yes
- ◯ No

Automatic Writing Rating

20. Please rate your Automatic Writing ability.

On a scale of low (left) to high (right)....
Strength -How intensely do you experience this ability?
Accuracy- How correct is the information?
Frequency- How often do you experience this ability?

	Weak/Inaccurate/Rarely				Strong/Accurate/Often
Strength	◯	◯	◯	◯	◯
Accuracy	◯	◯	◯	◯	◯
Frequency	◯	◯	◯	◯	◯

Astral Projection Ability

21. Astral Projection (or astral travel)- An out-of-body experience in which the "astral body" separates from the physical body and is capable of travelling outside it.

- ○ Yes
- ○ No

Astral Projection Rating

22. Please rate your Astral Projection ability.

On a scale of low (left) to high (right)....
Strength -How intensely do you experience this ability?
Accuracy- How correct is the information?
Frequency- How often do you experience this ability?

	Weak/Inaccurate/Rarely				Strong/Accurate/Often
Strength (intensity of projection)	◯	◯	◯	◯	◯
Accuracy (clarity of projection)	◯	◯	◯	◯	◯
Frequency	◯	◯	◯	◯	◯

Channeling Ability

23. Channel- Communication of information to or through a physically embodied human being from a non-physical source.

- ◯ Yes
- ◯ No

Channeling Rating

24. Please rate your Channeling ability.

On a scale of low (left) to high (right)....
Strength -How intensely do you experience this ability?
Accuracy- How correct is the information?
Frequency- How often do you experience this ability?

	Weak/Inaccurate/Rarely				Strong/Accurate/Often
Strength	◯	◯	◯	◯	◯
Accuracy	◯	◯	◯	◯	◯
Frequency	◯	◯	◯	◯	◯

Psychophony Ability

25. Psychophony- Speaking under the influence of a spirit.

- ◯ Yes
- ◯ No

Psychophony Rating

26. Please rate your Psychophony ability.

On a scale of low (left) to high (right)....
Strength -How intensely do you experience this ability?
Accuracy- How correct is the information?
Frequency- How often do you experience this ability?

	Weak/Inaccurate/Rarely				Strong/Accurate/Often
Strength	○	○	○	○	○
Accuracy	○	○	○	○	○
Frequency	○	○	○	○	○

Clairvoyance Ability

27. Clairvoyance or Extrasensory perception (ESP) - Clear vision, to visually perceive using the “mind's eye.”

- ◯ Yes
- ◯ No

Clairvoyant Rating

28. Please rate your Clairvoyant ability.

On a scale of low (left) to high (right)....
Strength -How intensely do you experience this ability?
Accuracy- How correct is the information?
Frequency- How often do you experience this ability?

	Weak/Inaccurate/Rarely				Strong/Accurate/Often
Strength	○	○	○	○	○
Accuracy	○	○	○	○	○
Frequency	○	○	○	○	○

Clairaudience Ability

29. Clairaudience- Clear audio/hearing, to hear from sources broadcast from spiritual or ethereal realm using the “inner ear.”

- ◯ Yes
- ◯ No

Clairaudience Rating

30. Please rate your Clairaudience ability.

On a scale of low (left) to high (right)....
Strength -How intensely do you experience this ability?
Accuracy- How correct is the information?
Frequency- How often do you experience this ability?

	Weak/Inaccurate/Rarely				Strong/Accurate/Often
Strength	○	○	○	○	○
Accuracy	○	○	○	○	○
Frequency	○	○	○	○	○

Clairsentience Ability

31. Clairsentience- Clear sensation or feeling within the whole body without any outer stimuli related to the feeling or information.

- ◯ Yes
- ◯ No

Clairsentience Rating

32. Please rate your Clairsentience ability.

On a scale of low (left) to high (right)....
Strength -How intensely do you experience this ability?
Accuracy- How correct is the information?
Frequency- How often do you experience this ability?

	Weak/Inaccurate/Rarely				Strong/Accurate/Often
Strength	◯	◯	◯	◯	◯
Accuracy	◯	◯	◯	◯	◯
Frequency	◯	◯	◯	◯	◯

Clairscent Ability

33. Clairscent- Clear smelling, To smell a fragrance/odor of substance or food which is not in one's surroundings.

- ◯ Yes
- ◯ No

Clairscent Rating

34. Please rate your Clairscent ability.

On a scale of low (left) to high (right)....
Strength -How intensely do you experience this ability?
Accuracy- How correct is the information?
Frequency- How often do you experience this ability?

	Weak/Inaccurate/Rarely				Strong/Accurate/Often
Strength	○	○	○	○	○
Accuracy	○	○	○	○	○
Frequency	○	○	○	○	○

Clairgustance Ability

35. Clairgustance- Clear tasting, to taste without putting anything in one's mouth.

- ◯ Yes
- ◯ No

Clairgustance Rating

36. Please rate your Clairgustance ability.

On a scale of low (left) to high (right)....
Strength -How intensely do you experience this ability?
Accuracy- How correct is the information?
Frequency- How often do you experience this ability?

	Weak/Inaccurate/Rarely				Strong/Accurate/Often
Strength	○	○	○	○	○
Accuracy	○	○	○	○	○
Frequency	○	○	○	○	○

Clairempathy Ability

37. Clairempathy- Clear emotion to feel emotions of another person or non-physical entity (also known as empath).

- ◯ Yes
- ◯ No

Clairempathy Rating

38. Please rate your Clairempathy ability.

On a scale of low (left) to high (right)....
Strength -How intensely do you experience this ability?
Accuracy- How correct is the information?
Frequency- How often do you experience this ability?

	Weak/Inaccurate/Rarely				Strong/Accurate/Often
Strength	○	○	○	○	○
Accuracy	○	○	○	○	○
Frequency	○	○	○	○	○

Claircognizance Ability

39. Claircognizance or Knowing - the empathic ability to feel what needs to be done in any given circumstance, often accompanied by a feeling of peace and calm, even in the midst of a crisis. Having the ability to understand or know something without any direct evidence or reasoning process.

- ◯ Yes
- ◯ No

Claircognizance Rating

40. Please rate your Claircognizance ability.

On a scale of low (left) to high (right)....
Strength -How intensely do you experience this ability?
Accuracy- How correct is the information?
Frequency- How often do you experience this ability?

	Weak/Inaccurate/Rarely				Strong/Accurate/Often
Strength	○	○	○	○	○
Accuracy	○	○	○	○	○
Frequency	○	○	○	○	○

Emotional Healing Ability

41. Emotional Healing - The empathic ability to feel another person's emotions (and often the ability to heal, transform or transmute them).

- ◯ Yes
- ◯ No

Emotional Healing Rating

42. Please rate your Emotional Healing ability.

On a scale of low (left) to high (right)....
Strength -How intensely do you experience this ability?
Accuracy- How correct is the information?
Frequency- How often do you experience this ability?

	Weak/Inaccurate/Rarely				Strong/Accurate/Often
Strength	○	○	○	○	○
Accuracy	○	○	○	○	○
Frequency	○	○	○	○	○

Geomancy Ability

43. Geomancy - The empathic ability to read the energy of places and of the land such as Ley lines.

- ◯ Yes
- ◯ No

Geomancy Rating

44. Please rate your Geomancy ability.

On a scale of low (left) to high (right)....
Strength -How intensely do you experience this ability?
Accuracy- How correct is the information?
Frequency- How often do you experience this ability?

	Weak/Inaccurate/Rarely				Strong/Accurate/Often
Strength	◯	◯	◯	◯	◯
Accuracy	◯	◯	◯	◯	◯
Frequency	◯	◯	◯	◯	◯

Levitation Ability

45. Levitation- to float in the air, defying gravity.

◯ Yes

◯ No

Levitation Rating

46. Please rate your Levitation ability.

On a scale of low (left) to high (right)....
Strength -How intensely do you experience this ability?
Accuracy- How correct is the information?
Frequency- How often do you experience this ability?

	Weak/Inaccurate/Rarely				Strong/Accurate/Often
Strength	○	○	○	○	○
Accuracy	○	○	○	○	○
Frequency	○	○	○	○	○

Lucid Dreamer Ability

47. Lucid dreamer – Ability to have awareness while dreaming. Knowing that you are dreaming while asleep.

- ◯ Yes
- ◯ No

Lucid Dreaming Rating

48. Please rate your Lucid Dreaming ability.

On a scale of low (left) to high (right)....
Strength -How intensely do you experience this ability?
Accuracy- How correct is the information?
Frequency- How often do you experience this ability?

	Weak/Inaccurate/Rarely				Strong/Accurate/Often
Strength	○	○	○	○	○
Accuracy	○	○	○	○	○
Frequency	○	○	○	○	○

Mediumship Ability

49. Mediumship- To mediate communication between spirits of the dead and living; the empathic ability to feel the presence and energies of spirits.

- ◯ Yes
- ◯ No

Mediumship Rating

50. Please rate your Mediumship ability.

On a scale of low (left) to high (right)....
Strength -How intensely do you experience this ability?
Accuracy- How correct is the information?
Frequency- How often do you experience this ability?

	Weak/Inaccurate/Rarely				Strong/Accurate/Often
Strength	○	○	○	○	○
Accuracy	○	○	○	○	○
Frequency	○	○	○	○	○

Nature Empath Ability

51. Nature empath- The empathic ability to read, feel and communicate with nature and with plants.

- ◯ Yes
- ◯ No

Nature Empath Rating

52. Please rate your Nature Empathic ability.

On a scale of low (left) to high (right)....
Strength -How intensely do you experience this ability?
Accuracy- How correct is the information?
Frequency- How often do you experience this ability?

	Weak/Inaccurate/Rarely				Strong/Accurate/Often
Strength	○	○	○	○	○
Accuracy	○	○	○	○	○
Frequency	○	○	○	○	○

Physical Healing

53. Physical Healing - The empathic ability to feel other people's physical symptoms in your own body (and often the ability to heal, transform or transmute them).

- ○ Yes
- ○ No

Physical Healing Rating

54. Please rate your Physical Healing ability.

On a scale of low (left) to high (right)....
Strength -How intensely do you experience this ability?
Accuracy- How correct is the information?
Frequency- How often do you experience this ability?

	Weak/Inaccurate/Rarely				Strong/Accurate/Often
Strength	◯	◯	◯	◯	◯
Accuracy	◯	◯	◯	◯	◯
Frequency	◯	◯	◯	◯	◯

Precognition Ability

55. Precognition, premonition and precognitive dreams - A form of clairvoyance when the objects of perception is distant in time; Perception of events before they happen; the empathic ability to feel when something important is about to happen (often this can be a feeling of inexplicable dread or doom).

- ◯ Yes
- ◯ No

Precognition Rating

56. Please rate your Precognition ability.

On a scale of low (left) to high (right)....
Strength -How intensely do you experience this ability?
Accuracy- How correct is the information?
Frequency- How often do you experience this ability?

	Weak/Inaccurate/Rarely				Strong/Accurate/Often
Strength	◯	◯	◯	◯	◯
Accuracy	◯	◯	◯	◯	◯
Frequency	◯	◯	◯	◯	◯

Psychic Surgery Ability

57. Psychic Surgery- Removal of diseased body tissue via an incision that heals immediately afterwards.

- ◯ Yes
- ◯ No

Psychic Surgery Rating

58. Please rate your Psychic Surgery ability.

On a scale of low (left) to high (right)....
Strength -How intensely do you experience this ability?
Accuracy- How correct is the information?
Frequency- How often do you experience this ability?

	Weak/Inaccurate/Rarely				Strong/Accurate/Often
Strength	◯	◯	◯	◯	◯
Accuracy	◯	◯	◯	◯	◯
Frequency	◯	◯	◯	◯	◯

Psychokinesis Ability

59. Psychokinesis or telekinesis - The ability to manipulate objects by the power of thought.

- Yes
- No

Psychokinesis Rating

60. Please rate your Psychokinesis ability.

On a scale of low (left) to high (right)....
Strength -How intensely do you experience this ability?
Accuracy- How correct is the information?
Frequency- How often do you experience this ability?

	Weak/Inaccurate/Rarely				Strong/Accurate/Often
Strength	○	○	○	○	○
Accuracy	○	○	○	○	○
Frequency	○	○	○	○	○

Psychometry Ability

61. Psychometry or psychoscopy or clairtangency- Clear touching; Obtaining information by touching or concentrating on an object; the empathic ability to receive energy, information and impressions from objects, photographs or places.

- ◯ Yes
- ◯ No

Psychometry Rating

62. Please rate your Psychometry ability.

On a scale of low (left) to high (right)....
Strength -How intensely do you experience this ability?
Accuracy- How correct is the information?
Frequency- How often do you experience this ability?

	Weak/Inaccurate/Rarely				Strong/Accurate/Often
Strength	○	○	○	○	○
Accuracy	○	○	○	○	○
Frequency	○	○	○	○	○

Pyrokinesis Ability

63. Pyrokinesis- The ability to create and/or manipulate fire through the concentration of mind.

- ◯ Yes
- ◯ No

Pyrokinesis Rating

64. Please rate your Pyrokinesis ability.

On a scale of low (left) to high (right)....
Strength -How intensely do you experience this ability?
Accuracy- How correct is the information?
Frequency- How often do you experience this ability?

	Weak/Inaccurate/Rarely				Strong/Accurate/Often
Strength	◯	◯	◯	◯	◯
Accuracy	◯	◯	◯	◯	◯
Frequency	◯	◯	◯	◯	◯

Remote Viewing Ability

65. Remote Viewing- The practice of seeking impressions about a distant or unseen target.

- ◯ Yes
- ◯ No

Remote Viewing Rating

66. Please rate your Remote Viewing ability.

On a scale of low (left) to high (right)....
Strength -How intensely do you experience this ability?
Accuracy- How correct is the information?
Frequency- How often do you experience this ability?

	Weak/Inaccurate/Rarely				Strong/Accurate/Often
Strength	○	○	○	○	○
Accuracy	○	○	○	○	○
Frequency	○	○	○	○	○

Retrocognition Ability

67. Retrocognition or post-cognition - Knowledge of a past event which could not have been learned or inferred by normal means.

- ◯ Yes
- ◯ No

Retrocognition Rating

68. Please rate your Retrocognition ability.

On a scale of low (left) to high (right)....
Strength -How intensely do you experience this ability?
Accuracy- How correct is the information?
Frequency- How often do you experience this ability?

	Weak/Inaccurate/Rarely				Strong/Accurate/Often
Strength	○	○	○	○	○
Accuracy	○	○	○	○	○
Frequency	○	○	○	○	○

Telepathy Ability

69. Telepathy- Communication of thoughts or ideas by means other than the known senses, mind-to-mind communication; the ability to read people's thoughts.

- ◯ Yes
- ◯ No

Telepathy Rating

70. Please rate your Telepathy ability.

On a scale of low (left) to high (right)....
Strength -How intensely do you experience this ability?
Accuracy- How correct is the information?
Frequency- How often do you experience this ability?

	Weak/Inaccurate/Rarely				Strong/Accurate/Often
Strength	○	○	○	○	○
Accuracy	○	○	○	○	○
Frequency	○	○	○	○	○

Family (A)

71. In general, at what age did your abilities start? (If the abilities started at different times of your life, please choose the first time you experienced any of the abilities.)

* 72. Does anyone else in your family have this or a different type of ability?

- Yes
- No

* 73. Are you a twin?

- Yes
- No

Family

74. Who in your family experiences extraordinary abilities and what abilities are they?
Please fill out all that apply to the best of your ability, leave extra rows blank if you have less than five members with abilities.

	Relation	Dominant ability	Age ability developed if known	Age now if known
Family member 1				
Family member 2				
Family member 3				
Family member 4				
Family member 5				

ability not listed (please specify family member and ability)

Experiences

75. Next you will read several statements about experiences you may have in your daily life. You will be asked how often you have these experiences, we are interested in how often these experiences happen to you when you are not under the influence of alcohol or drugs.

To answer the questions, please determine to what degree the experience described in the question applies to you and choose the button which corresponds to the percentage of the time you have the experience. The left of the scale, labeled ‘Never’, corresponds to 0% of the time, while the right of the scale, labeled ‘Always’, corresponds to 100% of the time; the range covers 0% to 100% in 10% increments.

	0%	10	20	30	40	50	60	70	80	90	100%
DT01. Some people have the experience of finding themselves in a place and having no idea how they got there.	○	○	○	○	○	○	○	○	○	○	○
DT02. Some people have the experience of finding new things among their belongings that they do not remember buying.	○	○	○	○	○	○	○	○	○	○	○
DT03. Some people sometimes have the experience of feeling as though they are standing next to themselves or watching themselves do something and they actually see themselves as though they were looking at another person.	○	○	○	○	○	○	○	○	○	○	○
DT04. Some people are told that they sometimes do not recognize friends or family members.	○	○	○	○	○	○	○	○	○	○	○
DT05. Some people sometimes have the experience of feeling that other people, objects, and the world around them are not real.	○	○	○	○	○	○	○	○	○	○	○
DT06. Some people sometimes have the experience of feeling that their body does not seem to belong to them.	○	○	○	○	○	○	○	○	○	○	○
DT07. Some people find that in one situation they may act so differently compared to another situation that they feel almost as if they were two different people.	○	○	○	○	○	○	○	○	○	○	○
DT08. Some people sometimes find that they hear voices inside their head which tell them to do things or comment on things that they are doing.	○	○	○	○	○	○	○	○	○	○	○

In the past three months have you experienced any of the following?

76. In the past three months have you experienced any of the following? Please indicate to what degree you have or have not experienced the items below and if you have experienced them, please rate how distressed (if at all) you were by this experience. If you marked "never" for an item, you can mark "does not apply" for the rating.

	Never	Sometimes	Often	Nearly Always	Not Distressed	A Bit Distressed	Quite Distressed	Very Distressed	Does Not Apply
PS01. Felt as if people seem to drop hints about you or say things with a double meaning?	☐	☐	☐	☐	☐	☐	☐	☐	☐
PS02. Felt as if some people are not what they seem to be?	☐	☐	☐	☐	☐	☐	☐	☐	☐
PS03. Felt that you are being persecuted in anyway?	☐	☐	☐	☐	☐	☐	☐	☐	☐
PS04. Felt as if there is a conspiracy against you?	☐	☐	☐	☐	☐	☐	☐	☐	☐
PS05. Felt that people look at you oddly because of your appearance?	☐	☐	☐	☐	☐	☐	☐	☐	☐
PS06. Felt as if electrical devices such as computers can influence the way you think?	☐	☐	☐	☐	☐	☐	☐	☐	☐
PS07. Felt as if the thoughts in your head are being taken away from you?	☐	☐	☐	☐	☐	☐	☐	☐	☐
PS08. Felt as if the thoughts in your head are not your own?	☐	☐	☐	☐	☐	☐	☐	☐	☐
PS09. Thoughts so vivid that you were worried other people would hear them?	☐	☐	☐	☐	☐	☐	☐	☐	☐
PS10. Heard your thoughts being echoed back at you?	☐	☐	☐	☐	☐	☐	☐	☐	☐
PS11. Felt as if you are under the control of some force or power other than yourself?	☐	☐	☐	☐	☐	☐	☐	☐	☐
PS12. Felt as if a double has taken the place of a family member, friend or acquaintance?	☐	☐	☐	☐	☐	☐	☐	☐	☐
PS13. Heard voices when you are alone?	☐	☐	☐	☐	☐	☐	☐	☐	☐
PS14. Heard voices talking to each other when you are alone?	☐	☐	☐	☐	☐	☐	☐	☐	☐
PS15. Seen objects, people or animals that other people can’t see?	☐	☐	☐	☐	☐	☐	☐	☐	☐

Medications

77. Do you take any psychoactive or psychiatric medications?

- ◯ Yes
- ◯ No

Medications

78. What medications are you taking?

	Medications
Medication 1	
Medication 2	
Medication 3	
Medication 4	
Medication 5	

Other (please specify)

Contact

* 79. May we contact you to continue participating in our study?

- ○ Yes
- ○ No

Contact Information

* 80. What is your name?

* 81. What is your email?

* 82. What is your phone number?

83. What is your preferred method of contact?

- Phone
- Email
- Other (please specify)

84. **THANK YOU FOR YOUR PARTICIPATION!**

If you said Yes to being contacted and left your contact information.
we may contact for the next phase of the research study.

Please go to **www.noetic.org** to learn more about the Institute of Noetic Sciences and our science projects.
