# Supplementary material for: People reporting experiences of mediumship have higher dissociation symptom scores than non-mediums, but below thresholds for pathological dissociation
Source: F1000Res. 2018 Jan 4;6:1416. Originally published 2017 Aug 10. [Version 3] doi: 10.12688/f1000research.12019.3 (PMC5782403; doi:10.12688/f1000research.12019.3)
Supplement: Peer review history

# Peer review: Referee response for version 3

**DOI:** 10.5256/f1000research.14736.r30021

**Published:** 2018-01-19

**Authors:** Adrian Parker

**Affiliations:** 1 Department of Psychology, University of Gothenburg, Gothenburg, Sweden

The authors have taken into account the mandatory changes. The article is now substantially improved and merits indexing.

I have read this submission. I believe that I have an appropriate level of expertise to confirm that it is of an acceptable scientific standard.

# Peer review: Referee response for version 2

**DOI:** 10.5256/f1000research.13998.r27912

**Published:** 2017-12-01

**Authors:** Adrian Parker

**Affiliations:** 1 Department of Psychology, University of Gothenburg, Gothenburg, Sweden

The version I have seen is basically sound but needs some attention to the issues that I raise below. As a native (UK) English speaker, I can endorse that the English in this version is correct (with the exception of a couple of missing words noted below) and clear. The statistical treatments are appropriate and are judged to be correct. The study has some importance relating to the efforts to distinguish little understood spiritual and transpersonal experiences from the more bizarre and perhaps pathological ones.

 Given the major constraints of the word count then the paper is acceptable but it is less than commendable that no hint of the complexity of the issues has been made in the paper. The article focuses rather exclusively on one singular conception of dissociation – that by Colin Ross and co-workers and their the DES-T scale. According to this view there is a normal continuum to dissociative experiences along with a cut-off point for the more bizarre types of experiences, which are seen then as an expression of pure pathology. The eight questions from DES-T that were selected for this study are those highlighting this potential diagnosis. There are of course other conceptions – such as those of Watson and co-workers - and at least a half dozen other tests of dissociation (Watson, 2001 ^1^; Watson & Loftus, 1999 ^2^). The authors write “Claims of such abilities have been considered symptoms of dissociative disorders” which is partially true but it is equally true that contact with dead (or even living) spirits is often considered to be symptomatic of individuals being psychosis-prone that is schizotypy – and if there is no distress to warrant a main DSM-diagnosis or DSM axis of personality disturbance - then this is labeled “happy schizotypy”. Indeed, found amongst the authors’ battery of tests and questions which number some 70 or more, are a set relating to just this. From this presentation it is clear that this study is only a minor part of a larger one, which will be or is being ”milked” for several papers. This is of course nowadays a standard practice (but involving naturally some major weaknesses) with internet studies. What is perhaps here rather misleading is that the study is presented to the potential responders as ”a study of genetics of extended human experience” – a presentation which might be considered a little disingenuous in the context of the issues in the current paper(s).

 This brings up the issue of what I think needs to be dealt with before indexing: how the context of the presentation might have influenced the major finding that the authors report and which the reviewer Prof. Ross attributes much importance to, namely that mediumship claimants were twice as likely as non-claimants to end up on the ill-side of the pathology cut-off. Although together 42% of the respondents endorsed communicating with the dead - the so-called ”AIR experiences”, it should be emphasized that AIR experiences were not the same as trance in that so-called channeling and clairaudience experiences might fall under this. Indeed it is surprising that a simple question concerning trance states does not seem to have been included since such individuals might have formed an interesting core group. The Noetic Sciences website under the aegis of which the (Surveymonkey) study was conducted, is dedicated to studying ”interconnectiveness” so it seems likely that those viewing the website and willing to answer the huge multitude of questions were highly motivated to confirm the mission of the website. This may well have created response biases. The authors write: ”Notably, the top five endorsed DES-T items were consistent with an AIR experience.” (Personally I think the addition of yet another acronym and neologism in the form of AIR only adds confusion to the overladen terminology in this field – it would surely have been better to keep to the explicit term mediumhip.). Accordingly I think their comment should be extended here so as to make some concession as to how the above findings can be vulnerable to the above mentioned influence.

 Page 1 line 6 from bottom: should read  than non-claimants

 Page 3 line 3 from bottom should read (presumably) of pathological dissociative

I have read this submission. I believe that I have an appropriate level of expertise to confirm that it is of an acceptable scientific standard, however I have significant reservations, as outlined above.

## References

[1] WatsonD: Dissociations of the night: individual differences in sleep-related experiences and their relation to dissociation and schizotypy. *J Abnorm Psychol*.2001;110(4) :526-35 1172794210.1037//0021-843x.110.4.526

[2] WrightDBLoftusEF: Measuring dissociation: comparison of alternative forms of the dissociative experiences scale. *Am J Psychol*.1999;112(4) :497-519 10696264

# Peer review: Referee response for version 2

**DOI:** 10.5256/f1000research.13998.r27178

**Published:** 2017-11-06

**Authors:** Colin A. Ross

**Affiliations:** 1 The Colin A. Ross Institute for Psychological Trauma, Richardson, TX, USA

This is an interesting study with a large N. Within the limitations of the methodology, it tells us that individuals with anomalous information reception (AIR) tend to be somewhat more dissociative than those without such experiences. This is consistent with prior literature. The study would have been stronger if the taxometric statistical analysis had been applied to the 8 DES-T items (available at www.isst-d.org), and if the full 28-item DES had been administered, but this would have made for a lengthy survey. The sample population is more dissociative and has more AIR than the general population and this provides interesting information about IONS members and affiliates that I don’t think has been published before. As Dr. Cardena noted, the references could have been more extensive, but I would say they are sufficient for a brief report.

I have read this submission. I believe that I have an appropriate level of expertise to confirm that it is of an acceptable scientific standard.

# Peer review: Referee response for version 1

**DOI:** 10.5256/f1000research.12999.r24906

**Published:** 2017-08-21

**Authors:** Etzel Cardeña

**Affiliations:** 1 Center for Research on Consciousness and Anomalous Psychology (CERCAP), Department of Psychology, Lund University, Lund, Sweden

This paper is a good example of why the F1000Research model is so bad... The manuscript is poorly written, does not show a good grasp of the relevant literature or that a good literature search was conducted, misrepresents some of its references, and very probably has an important mistake in a Table or its analyses. All of these issues could have been solved during the regular peer review process so that at the end the only publicly available version would have been an adequate one.

 Although it is a short paper, it would take me too long to list all of the problems in it, so I will just mention 2-3 examples per problematic issue:

1. Poor writing:
  From the abstract: a) "symptoms of [a] dissociation [dissociative] disorder". b) "Both AIR claimants and non-claimants scored lower than the clinical cutoff" [despite the previous sentence in the abstract mentioning that percentages of both groups had scored above the cutoff. c) "incorporating [a] comprehensive dissociative symptom measurement, as well as their effects on the person's functionality" [grammatical number is inconsistent, besides the fact that "measurement[s]" would not have an effect on functionality.
2. Inadequate coverage of the literature:
  a) There have been various recent studies specifically evaluating possible psychopathology in people reporting anomalous experiences (in general) and spirit possession/mediumship (in particular), yet only very few are listed in the Reference section. b) Contrary to what the authors write that "Claims of such abilities are often considered to be symptoms of dissociation [dissociative] disorders", yet both the anthropological literature and, more relevant in this case, the Diagnostic and Statistical Manual taxonomy, ever since its 4th edition, has specifically required that clinically significant levels of distress or dysfunction be present to consider a dissociative manifestation pathological.
3. Misrepresentations of cited literature:
  a) A paper by Rebecca Seligman is used to support the above quotation that mediumship abilities are often considered to be symptoms of dissociation, yet she specifically states that "dissociation is not a pathological experience, but rather a therapeutic mechanism", along the lines of what others in anthopology and psychology have written. b) "Almost half of United States adults...", yet this study was conducted in Winnipeg, Canada.
4. Statistical issues:
  a) In Table 1, a 3% difference (87 vs 84%) is reported as significant at the minus .05 level, yet an almost identical difference with about the same number of participants (87 vs 89%) is reported as non-significant. I very much doubt that both statements, particularly the first one, are accurate. b) There are multiple references to probability values = 0 or less than 0, but of course it goes against inferential statistics to state that instead of, for example, less than .001, or whatever.

I have read this submission. I believe that I have an appropriate level of expertise to state that I do not consider it to be of an acceptable scientific standard, for reasons outlined above.

# Response

**Published:** 2017-10-10

**Authors:** Helané Wahbeh

**Affiliations:** Oregon Health & Science University, USA

Response to Reviewer #1

 "This paper is a good example.....so that at the end the only publicly available version would have been an adequate one."

 - *Thank you for you taking the time to review our paper and for your thoughtful comments. Yes, we agree that through the traditional peer-review process, the final public version is superior to initial versions. We appreciate the transparent nature of F1000 review process as a way to reduce bias in publishing.*

 "Poor writing"

 - *Thank you for highlighting writing errors in our manuscripts. We have corrected the highlighted grammatical errors and unclear wording. We have also reviewed the entire paper for other writing errors. * 

 "Inadequate coverage of the literature"

*-We agree that the paper does not fully examine the literature related to this topic. The reason for this is the word count limits of a short article (1000 words). We have included additional references and attempted to succinctly include a broader understanding of the current literature.*

 "Misrepresentations of cited literature"

 a) A paper by Rebecca Seligman

*- Rebecca Seligman also states “Medical approaches to the question have implicated psychological disturbance as a motivational factor for some, yet they fail to explain how and why some psychological disturbances, in some individuals, come to be expressed as spirit-possession mediumship.” She also has a very nice discussion in the section “MEDIUMSHIP AND MENTAL ILLNESS REVISITED” on mediumship being considered a pathology. Because of the word limitations, we did not highlight the specific sections of her paper that refer to these issues but allow the reader to read the paper to appreciate the full scope of the discussion of mediumship and mental illness.*

 b) "Almost half of United States adults...", yet this study was conducted in Winnipeg, Canada.

*-This reference has been corrected.*

 "Statistical issues"

 a) In Table 1, a 3% difference (87 vs 84%) ..... I very much doubt that both statements, -particularly the first one, are accurate."

 - *These statistics have been double checked and they are accurately represented. We have also included a conservative Bonferroni multiple comparisons correction.*

**

 b) There are multiple references to probability values = 0 or less than 0, but of course it goes against inferential statistics to state that instead of, for example, less than .001, or whatever.

*-The p statistic reporting has been corrected.*
